# Promoting Reminiscences with Virtual Reality Headsets: A Pilot Study with People with Dementia

**DOI:** 10.3390/ijerph17249301

**Published:** 2020-12-12

**Authors:** Tiago Coelho, Cátia Marques, Daniela Moreira, Maria Soares, Paula Portugal, António Marques, Ana Rita Ferreira, Sónia Martins, Lia Fernandes

**Affiliations:** 1 Department of Occupational Therapy, School of Health, Polytechnic of Porto, R. Dr. António Bernardino de Almeida 400, 4200-072 Porto, Portugal; ppc@ess.ipp.pt (P.P.); ajmarques@ess.ipp.pt (A.M.); 2 Psychosocial Rehabilitation Lab, Center for Rehabilitation Research (CIR), R. Dr. António Bernardino de Almeida 400, 4200-072 Porto, Portugal; 3 Center for Health Technology and Services Research (CINTESIS), Faculty of Medicine, University of Porto, R. Dr. Plácido da Costa, 4200-450 Porto, Portugal; up201309446@med.up.pt (A.R.F.); soniamartins@med.up.pt (S.M.); lfernandes@med.up.pt (L.F.); 4 Occupational Therapy Course, School of Health, Polytechnic of Porto, R. Dr. António Bernardino de Almeida 400, 4200-072 Porto, Portugal; catiacm97@gmail.com (C.M.); danielamoreira.6.dm@gmail.com (D.M.); mils1708@gmail.com (M.S.); 5 Department of Clinical Neurosciences and Mental Health, Faculty of Medicine, University of Porto, Alameda Prof. Hernâni Monteiro, 4200-319 Porto, Portugal; 6 Psychiatry Service, Centro Hospitalar Universitário de São João (CHUSJ), Alameda Prof. Hernâni Monteiro, 4200-319 Porto, Portugal

**Keywords:** dementia, reminiscences, virtual reality

## Abstract

This study aimed to explore the feasibility and effects of promoting reminiscences, using virtual reality (VR) headsets for viewing 360° videos with personal relevance, with people with dementia. A study with a mixed methods design was conducted with nine older adults diagnosed with dementia. Interventions consisted of four sessions, in which the participants’ engagement, psychological and behavioral symptoms, and simulation sickness symptoms were evaluated. Neuropsychiatric symptomatology and quality of life were measured pre- and post-intervention. Caregivers were interviewed regarding the effect of the approach. In most cases, participants appeared to enjoy the sessions, actively explored the 360° environment, and shared memories associated with the depicted locations, often spontaneously. There were no cases of significant increases in simulator sickness and psychological and behavioral symptoms during sessions, with only some instances of minor eyestrain, fullness of head, anxiety, irritability, and agitation being detected. Although there were no significant changes in the measured outcomes after intervention, the caregivers assessed the experience as potentially beneficial for most participants. In this study, promoting reminiscences with VR headsets was found to be a safe and engaging experience for people with dementia. However, future studies are required to better understand the added value of immersion, using VR, in reminiscence therapy.

## 1. Introduction

Dementia is a syndrome that is characterized by cognitive decline (affecting domains such as memory, language, orientation, attention and judgment), psychological and behavioral symptoms (such as depression, anxiety, apathy, disinhibition and irritability), and disability in daily activities [1,2]. It is usually caused by neurodegenerative diseases, such as Alzheimer’s disease, which accounts for 60–70% of cases [3]. While there are potentially modifiable risk factors for dementia (such as low education, excessive alcohol consumption, obesity, social isolation and physical inactivity), age is the strongest known risk factor [3,4]. Consequently, as longevity increases globally, so does the number of people living with dementia [4]. There are currently more than 50 million people with dementia, an amount estimated to grow to 152 million by the year 2050 [5]. Therefore, considering that dementia is a major cause of disability and healthcare utilization, impacting not only individuals but also their families and societies, research and innovation regarding the prevention and treatment of the syndrome should be a public health priority [3,6].

Current recommendations about the treatment of psychological and behavioral symptoms of dementia focus on non-pharmacological interventions [7,8]. Some of these interventions take into consideration the patients’ background and life narrative to develop tailored approaches [9,10,11]. Reminiscence therapy is a well-supported example of these methodologies, consisting of a psychosocial intervention that involves the recollection and discussion of past personal experiences, usually with the aid of prompts such as photos, videos, music or objects [7,9]. As people with dementia are usually able to recall more events from their early life than recent experiences, interventions that focus on preserved remote autobiographical memories are particularly relevant [9]. Research shows that promoting reminiscences with people with dementia may lead to improvements in communication, quality of life, and psychological and behavioral symptoms such as depression and agitation [7,9]. However, the evidence regarding reminiscence interventions with immersive cues—such as permitted by virtual reality (VR)—is still sparse.

VR is often used for recreational purposes, but also increasingly in therapeutic settings, with goals such as addressing phobias, improving knowledge and empathy regarding a condition, and developing motor or cognitive skills [12,13,14,15]. VR systems promote mostly audiovisual experiences and allow for different degrees of immersion, ranging from the non-immersive (in which, for example, visual stimuli are presented on a monitor) to the fully immersive (using technologies such as head-mounted displays, also known as VR headsets or goggles) [14,16]. Immersion can be achieved when a vivid and surrounding experience is presented, occupying the individual’s field of view while significantly reducing stimuli from physical reality, which in turn may lead to a sense of presence in the virtual environment [15,16]. Current research suggests that immersive VR is safe, well-tolerated and capable of promoting engagement and providing pleasant experiences to people with dementia [12,13,14,17,18,19,20,21,22]. Furthermore, a recent study by Rose et al. [17], using VR headsets, has shown that when participants with dementia observed 360° videos of locations that looked familiar, they often would reminisce about the past.

VR headsets can be used to observe computer-generated digital environments as well as 360° video footage. As Slater and Sanchez-Vives [15] describe, these are different possibilities that fit in the VR domain, with distinct strengths and limitations. Indeed, 360° videos currently do not allow to freely change the viewpoint or to interact with the environment. However, they are easier to create—due to the increasing accessibility of 360° cameras—and they depict real recognizable settings, which can be vital for reminiscence therapy, as higher realism of VR stimuli can facilitate the recollection of autobiographical memories [23,24].

Considering that little research has focused on the topic, the goals of the present pilot study are (A) to evaluate the feasibility of promoting reminiscences with people with dementia using VR headsets for viewing 360° video recordings of locations that are relevant to each individual’s life story; (B) to explore the effects of a brief reminiscence program, consisting of four individual sessions using the described methodology, in the psychological and behavioral symptoms and quality of life of people with dementia; and (C) to understand the perspectives of the participants’ caregivers regarding the impact of the program on the well-being of the participants.

## 2. Materials and Methods

### 2.1. Study Design and Sample

A pilot study was conducted with a mixed methods design. The feasibility of the VR approach to reminiscence therapy was assessed by examining the participants’ engagement, the expression of the psychological and behavioral symptoms associated with dementia, and the exacerbation of symptoms related with simulation experiences during the sessions. The effects of the reminiscence program were analyzed by a simple comparison of overall psychological and behavioral symptomatology and quality of life, between pre- and post-intervention. Finally, the caregivers’ perspective was examined with a qualitative approach, by analyzing the content of transcriptions resulting from semi-structured interviews conducted after the intervention.

A convenience sample, consisting of nine individuals with dementia, was recruited in two local institutions that provide health and social services to older adults in the district of Porto, Portugal. Inclusion criteria consisted of being aged ≥65 years and having a clinical diagnosis of dementia. Exclusion criteria were severe visual deficits (since it could interfere significantly with the visualization of the videos), inability to verbally communicate (as communication was required for selected measurements), classification as being in stages 1–3 of the Global Deterioration Scale [25,26] (considered as pre-dementia stages), diagnosis of Lewy body dementia (due to higher probability of developing hallucinations [27]), being in a late stage of dementia (evaluated as in the seventh stage of the Global Deterioration Scale, due to very severe cognitive deficits and associated limitations), and diagnosis of other psychiatric disorders (including schizophrenia, schizoaffective disorder, delusional disorder, psychotic disorders not otherwise specified, bipolar disorder, or major depressive disorder, in order to reduce confounding effects). Participants had to have family members or other relevant individuals that could provide information about the participants’ life story. Additionally, it was required that participants had a caregiver who would be available to attend most of the reminiscence sessions and to participate in part of the assessments. In the present study, the term caregiver is used in a broader sense, including any individual who provided any kind of physical or emotional support to people with dementia, and spent a substantial part of their day, most days of the week, with the participants. Consequently, three caregivers (CG) were selected for this study: two professionals from the institutions (CG1, who was paired with five persons with dementia, and CG2, with three of the participants) and one family member (CG3, who accompanied his relative with dementia).

All participants, including individuals with dementia, informants who provided data about life stories, and caregivers, gave their written informed consent. The procedures of the present study conformed with the 1964 Helsinki Declaration and its later amendments, and the research was approved by the Ethical Committee of the School of Health of the Polytechnic of Porto (registry number: 5698).

### 2.2. Preparation and Implementation of the Reminiscence Program

The preparation of the intervention program involved conducting interviews with participants with dementia and with family members or other relevant individuals, in order to identify positive memories that could be depicted trough video recordings in 360°. A semi-structured interview guide was developed, comprising three main themes about the participants’ past: meaningful activities, settings and events. Before contacting the participants, this guide was analyzed by a panel of three researchers, experienced in gerontology, qualitative studies and life stories analysis, to validate the contents of the interview. Researchers charged with conducting the interviews underwent training about managing semi-structured interviews. Additionally, a preliminary interview was performed with a person with dementia and with a family member, with the goal of identifying if modifications were necessary. No changes were required, and the resulting guide was used in the interviews, which were conducted in the institutions where the participants were recruited. Duration of the interviews ranged from approximately 30 to 60 min. The transcriptions of the interviews were analyzed independently by two researchers, who were also tasked with identifying a set of five to six possible video recordings for each participant. Findings comprised mostly settings such as childhood houses and workplace locations, as well as leisure and religious venues. Consequently, the locations that were selected for filming included specific streets, squares, gardens, or churches with individual relevance, as well as historical landmarks that were meaningful to several participants (e.g., the Sanctuary of Bom Jesus, the Sanctuary of Fátima, or the Clérigos Tower).

A GoPro Fusion 360 camera was used to film the selected locations. The camera was placed on a tripod, with a height of 165 cm, in a position that ensured minimal interference with the mobility of people in that setting. Filming was conducted until there was between 20–30 min of recordings that did not include any unexpected interference (such as individuals or vehicles blocking the view) or any potentially stressful event (such as people arguing or dogs barking at the camera). When people passed closely by and in clear view of the camera, they were approached by researchers in order to obtain written consent to appear in the video. This was performed to reduce the need for blurring faces, which was avoided to prevent confusion in participants. GoPro Fusion Studio (GoPro, Inc., San Mateo, CA, USA) and Adobe Premiere Pro (Adobe Systems Software Ireland Limited, Dublin, Ireland) were used for video editing.

Intervention consisted of four individual reminiscence sessions, conducted over two weeks, in which participants viewed a 360° video recording of a location that was relevant to their life story, assisted by a previously trained researcher with experience in reminiscence therapy. Each session started with an initial preparation (including the assessment of simulator sickness symptoms and, if necessary, instructions about the equipment and ensuing procedures), followed by exposure to one of the videos (with a maximum duration of 15 min), and a final discussion (in which the researcher asked about how the participant was feeling and re-assessed simulator sickness symptoms). During the exposure, the researcher tasked with managing the session followed a semi-structured protocol consisting of verbal cues and questions, aimed at facilitating the exploration of the 360° environment and the recollection and discussion of associated memories, while allowing for some flexibility regarding the addressed subjects. This protocol included instructions and questions such as: “Please relax and look around you”; “Feel free to explore everything that surrounds you”; “What do you see?”; “Does this place make you remember anything?”; and “How does it make you feel?”. Exposure ended when the researcher felt that there were no additional aspects left to explore or when the participant asked to remove the VR headset. Average duration of exposure was approximately 10 min. Caregivers attended most sessions but had no interference during the intervention. Sessions took place where it was more convenient for participants, caregivers, and institutions. Consequently, part of the sessions occurred in the institutions where the participants were recruited, when there was a room available that could be used exclusively for the intervention. The remaining sessions took place in the Psychosocial Rehabilitation Lab of the Center for Rehabilitation Research (School of Health—Polytechnic of Porto), which has spaces dedicated for interventions with VR. Samsung Gear VR with a Samsung S7 smartphone and the Oculus Rift were the VR headsets used for the intervention. During the sessions, the participants sat in a rotating chair, facilitating the exploration of the 360° environment. Earphones were used but with very low volume, since it was required for participants to listen to the researcher’s prompts.

### 2.3. Data Collection

The data collected for the characterization of the participants consisted of sociodemographic variables (age, sex, marital status and education), disability in daily activities (obtained with the Barthel Index [28,29] and the Lawton and Brody Scale [30,31]), cognitive status (measured with the Montreal Cognitive Assessment [32,33]), and dementia stage (classified with the Global Deterioration Scale [25,26]). Sociodemographic characteristics and data related with disability were collected from participants and confirmed with caregivers. The Montreal Cognitive Assessment was directly administered to the participants and the Global Deterioration Scale was rated, having in consideration the previous assessments and information provided by caregivers and professionals from the institutions where the participants were recruited.

Regarding the measures, the Barthel Index [28,29] and the Lawton and Brody Scale [30,31] are used to rate disability in basic and instrumental activities of daily living, respectively. In both cases, lower scores represent higher disability. The Montreal Cognitive Assessment [32,33] is a brief cognitive screening test that evaluates several cognitive domains, including executive functions, memory, attention, language and orientation. Possible scores range from 0 to 30, with increased impairment resulting in lower scores. The Global Deterioration Scale [25,26] is used to classify the stage of progression of cognitive decline in dementia, and associated behavior and functional limitations. Stages range from 1—no cognitive decline, to 7—very severe cognitive decline.

Participants’ engagement and behavior during sessions were assessed trough observation, conducted separately by two researchers attending the intervention, using a scale developed for this study. After each session, researchers would reach a consensus regarding their assessments, with collaboration of the researcher that managed the session, if necessary. This scale comprised: level of interest in exploring the 360° environment (1—very interested, if the participants actively searched and looked for different parts of the environment; 2—moderately interested, if there was some exploration; and 3—not interested, if participants did not look around); communication (1—if participants communicated spontaneously several times, describing the video and/or associated memories without need for questions; 2—only after questions/prompts; or 3—did not communicate); degree of detail of described memories (1—in detail, 2—superficially, or 3—none); type of shared memories (1—mostly positive/happy memories, 2—shared equally positive and negative memories, and 3—mostly negative/sad memories); and assessment of the overall experience (1—pleasant, if the participant mostly appeared to enjoy the general experience, considering verbal and non-verbal information; 2—neutral, if there was no observable reaction to the experience; and 3—unpleasant, if the participant appeared to be unhappy during most of the experience). Researchers also took field notes to document additional relevant information.

A similar observational methodology was adopted to explore the manifestation of psychological and behavioral symptoms during the sessions. A scale was prepared, based on the Cornell Scale for Depression in Dementia [34,35] and the Neuropsychiatric Inventory [36,37], with symptoms rated as absent, intermittent/slight, moderate, or severe. It included depression (sad expression, sad voice, or tearfulness), anxiety (anxious expression, nervousness, rumination, or worrying), agitation (restlessness, hand wringing, or hair pulling), aggression (screams, verbally or physically tries to assault another person), euphoria (exaggeratedly happy, elated or highly communicative), apathy (apathetic reaction, absence of interest or lack of response to stimuli), disinhibition (acts impulsively or has an inadequate behavior), irritability (impatient or easily annoyed), delusion (expresses false beliefs), and hallucinations (appears to see, hear, or feel things that are not presented during the session).

As in previous studies with VR and people with dementia [38], specific symptoms associated with simulation experiences were assessed with the Simulator Sickness Questionnaire [39,40]. In this self-report questionnaire, which was administered to participants before and after exposure to VR by the researcher that managed the session, symptoms are rated as absent/none, slight, moderate, or severe. It includes: general discomfort, fatigue, headache, eyestrain, difficulty focusing, increased salivation, sweating, nausea, difficulty concentrating, fullness of head, blurred vision, dizzy (eyes open), dizzy (eyes closed), vertigo, stomach awareness and burping.

The overall psychological and behavioral symptomatology and the quality of life of the participants with dementia were assessed with the Neuropsychiatric Inventory [36,37] and with the EUROHIS-QOL-8 [41,42], respectively, pre- and post-intervention. The Neuropsychiatric Inventory [36,37] is administered as a structured interview with the caregiver or other knowledgeable informant, who report on the frequency and severity of 12 symptoms usually found in dementia patients. It produces a total score ranging from 0 to 144, with higher scores indicating higher frequency and severity of symptomatology. EUROHIS-QOL-8 is a brief self-report questionnaire, with scores ranging from 8 to 40 (higher scores are indicative of a better perceived quality of life).

Finally, interviews were conducted with caregivers after the last session of the intervention program, with the goal of understanding their opinion regarding the program and its impact on the well-being of the participants. A semi-structured interview guide was used, without pre-determined themes/categories. It included questions such as: “What do you think about the program?”; “What aspects do you remember about what you saw?”; “How to you describe the participant’s behavior?”; and “How do you describe the effects of the intervention for the participant?”. Before the interviews, a consultation with three experts in qualitative studies was conducted, to ascertain that the questions were adequately formulated. Interviews were transcribed verbatim.

All assessments were performed by trained researchers.

### 2.4. Data Analysis

Quantitative data are described using proportions, mean values and standard deviations, according to the nature of the variables. The Wilcoxon test was used to compare Neuropsychiatric Inventory and EUROHIS-QOL-8 scores, obtained before and after the intervention. Results were considered statistically significant if *p* < 0.05 (two-tailed). Version 24.0 of the IBM SPSS Statistics 24.0 (SPSS, Inc., Chicago, IL, USA) was used.

Qualitative content analysis of the transcriptions of the interviews consisted of an inductive thematic analysis [43], seeking an interpretation of data at a semantic level, focused on explicit meanings of the participants’ discourse. Excerpts of text were coded into main themes/categories, considering the assumptions of internal and external integrity (homogeneity of individual coded units within a category and differentiation between categories, respectively [44]). This process was conducted separately by two researchers, who subsequently met and discussed to achieve an agreement upon organization of the content.

## 3. Results

The participants’ mean age was 85.6 (±7.4) years and the majority were women (66.7%). The mean Montreal Cognitive Assessment score was 7.2 (±5.3) and there was an equal number of participants (33.3%) in stages 4, 5 and 6 of the Global Deterioration Scale. Additional characteristics of the participants are available in Table 1.

Most participants took part in the four sessions of the reminiscence program. However, one participant missed two sessions, due to general health problems, and one individual did not participate in the fourth session, as previous sessions were considered to be unpleasant. The assessment of the experience as negative by this participant resulted from self-reported difficulties in seeing and understanding parts of the environments displayed in the VR headset. These difficulties apparently resulted in some signs of mild agitation and irritability, with the participant asking to remove the headset shortly after the session started. However, during the entirety of the program, most experiences (83%) appeared to be pleasant for the participants and there were no cases of moderate or severe psychological and behavioral symptoms displayed during the exposure. Only a few cases of mild or intermittent symptoms were manifested, mainly regarding anxiety, agitated behavior and irritability. According to field notes, these symptoms were detected when participants had trouble understanding or recognizing particular details shown in the video (mostly distant visual stimuli such as words in posters or signs, as well as people’s faces) or when some negative/sad memories were described. Nevertheless, these mild symptoms occurred sparsely during the sessions, not interfering with the overall participation, except for the abovementioned individual.

In most cases (73.5%), participants were very interested in exploring the 360° environment, frequently pointing to certain parts and turning in the chair to survey their surroundings. Communication was frequently spontaneous (57.7%), without need for questions or prompts in order for the participants to describe what they were seeing and remembering. No participant remained silent during the experience and their discourse often included memories of their personal experiences associated with the locations displayed in the videos (71.1%). In most cases (56.2%), participants mainly addressed seemingly positive/happy memories.

Regarding simulator sickness, there were no cases of severe symptoms or significant escalation in symptomatology comparing pre- and post-session. There were a few cases of increase from none to slight, or from slight to moderate symptoms, particularly two cases of eyestrain and fullness of head, one case of blurred vision and one case of burping.

Further data regarding participants’ engagement, simulator sickness and psychological and behavioral symptoms displayed during reminiscence sessions are described in Table 2 and Table 3.

Comparing pre and post-intervention, no significant differences were found (*p* > 0.05) in Neuropsychiatric Inventory and in EUROHIS-QOL-8 scores (Table 4).

Regarding the qualitative analysis of the content of the caregivers’ interviews, two main categories/themes emerged: behavior displayed during sessions and overall impact of the intervention. All caregivers agreed that the use of VR headsets was mostly well-received by the participants and that it did not cause any significant discomfort or negative reaction during the sessions. Likewise, there was agreement that the participants enjoyed the general experience of viewing the 360° videos and talking about them (except for the participant that reported visual limitations). Two caregivers highlighted that, in their perspective, the participants were very motivated and communicative during the sessions (“*they liked and commented about what they saw (…) they pointed, they were curious (…) had fun and enjoyed the discovery.*” (CG1); “*it is great (…) they commented about that reality (…) everyone talked.*” (CG2)), whereas CG3 stated that there was no significant change in the usual behavior of the accompanied person with dementia. Considering some cases of mild anxiety or agitated behavior, CG1 considered that these were mainly caused by a disruption in the participants’ usual routine, particularly by asking them to move to a different location from what than they were used to in order to participate in the sessions. On the other hand, CG2 felt that some questions and verbal prompts used by the researcher during the visualization of the video caused some slight confusion in some participants (“*at a certain time it was asked «but where are you?» so I think this may confuse them. The fact that we are talking and not being seen.*”). Finally, CG1 and CG2 also highlighted that using an immersive approach might have promoted the engagement of the participants (“*the 360° video is more real (…) they perceive that they are in that space.*” (CG1); “*I noticed that it was more positive, the type of… involvement (…) it is immersive reality (…) which was different, indeed. That they benefited? They did.*” (CG2).

Regarding the impact of the intervention, in CG3′s opinion, there were no positive or negative changes after the intervention. In turn, both CG1 and CG2 claimed that the program was beneficial for the participants, at least for a few moments (“*I think that the general impact is positive.*” (CG1); “*in the next day, hours or minutes after, yes… after that, it faded (…) they were left with a good sensation after your presence, right? In that day at least (…) it worked (…) what happened in these few times is that they started asking things (…) I think that with this study we can show that people gain will, desire, and ambition again (…) we are making people live again*” (CG2)). Furthermore, in CG2′s opinion, this intervention program also resulted in an increased interest from the participants’ family in their relative’s life, as well in more frequent contacts between them, possibly due to the prior involvement of the family in the description of the life story of the person with dementia.

## 4. Discussion

The findings of the present study add to the evidence [12,13,14,17,18,19,20,21,27] that supports the safety and feasibility of using VR headsets to promote immersive experiences for people with dementia. Furthermore, results suggest that immersion with 360° videos, specifically of locations that are relevant to each individual’s life story, may lead people with dementia to reminisce and reenact stories from their past, often spontaneously. Consequently, the potential use of VR in reminiscence therapy is highlighted.

No significant adverse effects of the intervention were detected, regarding simulator sickness and psychological and behavioral symptoms. However, there were a few cases of minor increases in eyestrain, fullness of head, anxiety, irritability and agitation, during the reminiscence sessions. These mild neuropsychiatric symptoms may have resulted from several factors, such as the recollection of negative events (a potential risk in reminiscence therapy [9,45]), difficulty in perceiving certain details present in the video (e.g., distant faces or symbols), the disruption of the routine of the participants (a possible consequence of participating in a new and brief program of activities, since structured daily routines are important to manage behavioral symptoms [22,46]), or confusion caused by trying to communicate with the researcher, who was not visible (the experience involved the combination of visual stimuli displayed in the VR environment with auditory stimuli from the room where the session occurred). These factors, some of which were emphasized by caregivers who were present during most sessions, should be taken into account in future studies. The use of 360° videos with high resolution and devoid of small or distant components which may be hard to perceive should be prioritized. Furthermore, interventions should have minimal interference with the usual routine, contexts, and activities of people with dementia, to reduce the probability of the exacerbation of psychological and behavioral symptoms. Finally, researchers should assess if talking with participants while they are immerged in the VR environment—and unable to view the source of the auditory information—causes significant confusion or interferes with the sense of presence in the location. Concomitantly, alternatives should be considered, such as reducing or eliminating verbal interference during the immersive experience, or including, in the filming of video, a person that faces the camera and uses the necessary verbal and non-verbal prompts to facilitate the exploration of the environment, as well as the recollection and description of past events. These aspects could be important to enhance engagement during sessions, adherence to the intervention program, and potential positive outcomes.

In a study by Rose et al. [17], people with dementia reminisced about family, travels and geographical origins, when they observed a 360° video of a location that looked like a familiar place. The participants were able to choose a video from a pre-determined set of possibilities, including forests, beaches and a cathedral. In the present study, a more personalized approach was sought, involving a detailed collection of information regarding the participants’ life story, filming of locations that were relevant to each participant, and preparing a tailored program of sessions. This method aimed to ensure the reminiscence process, focusing on positive and happy memories, in order to improve the well-being of the participants. Most participants shared positive past experiences during the sessions (with varying levels of detail, which can be associated with the different videos that were used in each session and related memories, among other factors). However, this approach can be considered more strenuous, as it requires interviewing, traveling, and filming, taking into account the participants’ profile. This may limit the possibility of conducting similar interventions at a larger scale. Nevertheless, a more person-centered approach, which recognizes the individual’s biography, is more likely to promote engagement and well-being [47,48,49]. Furthermore, as observed in this study, different persons can have past experiences in the same locations, such as historical landmarks, which may reduce the quantity of distinct videos that are necessary for intervention programs. With the expanded availability of 360° videos online and of those resulting from projects of this nature, a considerable portfolio of videos is growing, which can be used in different therapeutic contexts. Additionally, the increasing affordability and portability of 360° cameras and VR headsets may facilitate similar approaches to reminiscence therapy.

In the present pilot study, there were no significant differences in the psychological and behavioral symptoms and in the quality of life of the participants, post-intervention. As described by Park et al. [7], evidence suggests that more than eight reminiscence sessions are required to obtain substantial therapeutic effects on these outcomes. However, the intervention in this study appeared to be pleasant for most individuals and caregivers felt that the participants enjoyed the sessions. Furthermore, the analysis of the caregivers’ discourse suggests that the intervention may have promoted communication and overall well-being in individuals with dementia. These outcomes are frequently observed in reminiscence trials [7,9], and should be considered in follow-up studies that examine the effects of this approach.

The recollection of places from the past is crucial for maintaining a sense of self, particularly in cases of dementia [49]. VR allows people with dementia to virtually travel to those places, which can lead to a sense of escapism associated with positive feelings [45]. It is argued that using 360° videos with VR headsets provides a high level of visual realism and immersion, which enhances the experience and facilitates the triggering of autobiographical memories [24,38]. However, future studies should be conducted to better understand the added value of using immersion in reminiscence therapy. Further research should also address the limitations of the present study, which include: small sample size; absence of control groups; short intervention program; inclusion of participants that did not attend all intervention sessions; inclusion of both formal and informal caregivers in the same group; variety of intervention settings; no follow-up assessment; no blinding of outcome assessment; lack of quality of life measures specifically targeted for cases of dementia (such as the Quality of Life in Alzheimer’s Disease Scale—QoL-AD [50]); lack of measures for specific components of the neuropsychiatric symptoms of dementia (such as depression and anxiety); as well as the lack of intervention outcomes related with cognition, communication, well-being, and caregiver burden. In future similar studies and interventions, having backup videos should also be considered, to use in case participants do not remember nor recognize a certain location, which is particularly relevant since the setting might have changed considerably over the years. Furthermore, considering the potential effects of immersive interventions in people with dementia, it is important that the scientific community discuss the applicability and ethical ramifications of similar approaches targeted at individuals in a late stage of dementia.

Despite the reported limitations, the present study has strengths that should be highlighted. The described approach to reminiscence therapy with VR has not been examined in detail in previous research. Furthermore, it constitutes a novel, person-centered, non-pharmacological intervention that could be used by several health professionals, in various settings. Additionally, the combination of different research methods and the involvement of distinct stakeholders (individuals with dementia, family members, and caregivers) could have contributed to a deeper understanding of the viability and impact of the proposed intervention approach. Additionally, the importance of using structured methods and protocols for preparing and conducting the intervention should be highlighted. Informed by the present results, we are designing a randomized-controlled trial with the goal of examining the effects of a reminiscence program consisting of a similar approach to the one described here, in comparison with a reminiscence program without immersive cues (using photos or presenting the same videos, for example, on a monitor), and with no intervention/treatment as usual.

## 5. Conclusions

The findings of the present study support the feasibility of promoting reminiscences with people with dementia using VR headsets and 360° videos of meaningful locations, considering that no significant detriment to the well-being of the participants was observed, and that participants were engaged and able to recall past events during the VR experience. Although there were no significant differences in psychological and behavioral symptoms and quality of life post-intervention, the reminiscence sessions, in most cases, appeared to be pleasant and enjoyed by the participants. Future studies are required to better examine the effects of this approach to reminiscence therapy, as non-pharmacological interventions should be adopted as first-line approaches to neuropsychiatric features of dementia and have been referred to as crucial to ensure the well-being of people with dementia and their families. Furthermore, the VR methodology examined in this study may constitute an alternative to physically traveling to locations that are associated with positive feelings and memories, which is particularly important when traveling is not possible due to lack of resources or safety restrictions.

## Figures and Tables

**Table 1:** Characteristics of the participants (*n* = 9).

Characteristics	*n* (%)
Sociodemographic characteristics	
Age (years), mean ± SD	85.6 ± 7.4
Sex	
Women	6 (66.7)
Men	3 (33.3)
Marital Status	
Unmarried/divorced/widowed	7 (77.8)
Married/living with partner	2 (22.2)
Education (completed school years), mean ± SD	5.8 ± 4.8
Disability in daily activities	
Barthel Index score (0–20), mean ± SD	14.2 ± 6.2
Lawton and Brody Scale score (0–23), mean ± SD	5.6 ± 5.1
Cognitive status	
Montreal Cognitive Assessment score (0–30), mean ± SD	7.2 ± 5.3
Global Deterioration Scale ^a^	
Stage 4—moderate cognitive decline	3 (33.3)
Stage 5—moderately severe cognitive decline	3 (33.3)
Stage 6—severe cognitive decline	3 (33.3)

^a^ Participants in stages 1–3 and 7 of the Global Deterioration Scale were not considered for inclusion in the present study.

**Table 2:** Participants’ engagement and behavior during reminiscence sessions.

Participants’ Engagement and Behavior During Sessions	Session 1 (*n* = 9)	Session 2 (*n* = 8)	Session 3 (*n* = 9)	Session 4 (*n* = 7)	Average % During the Program
	*n* (%)	*n* (%)	*n* (%)	*n* (%)	
Level of interest in exploring the 360° environment					
Very interested	6 (66.7)	6 (75.0)	6 (66.7)	6 (85.7)	73.5
Moderately interested	2 (22.2)	2 (25.0)	3 (33.3)	1 (14.3)	23.7
Not interested	1 (11.1)				2.8
Communication					
Communicated spontaneously	4 (44.4)	5 (62.5)	6 (66.7)	4 (57.1)	57.7
Communicated after questions/prompts	5 (55.6)	3 (37.5)	3 (33.3)	3 (42.9)	42.3
Did not communicate					
Degree of detail of described memories					
Described memories in detail	4 (44.4)	4 (50.0)	3 (33.3)	2 (28.6)	39.1
Described memories superficially	1 (11.1)	3 (37.5)	2 (22.2)	4 (57.1)	32.0
Did not share any memories	4 (44.4)	1 (12.5)	4 (44.4)	1 (14.3)	28.9
Type of shared memories ^a^					
Mostly positive/happy memories	3 (60.0)	5 (71.4)	3 (60.0)	2 (33.3)	56.2
Positive and negative	2 (40.0)	1 (14.3)	1 (20.0)	4 (66.7)	35.2
Mostly negative/sad memories		1 (14.3)	1 (20.0)		8.6
Assessment of the overall experience					
Pleasant	6 (66.7)	7 (87.5)	7 (77.8)	7 (100.0)	83.0
Neutral	3 (33.3)		1 (11.1)		11.1
Unpleasant		1 (12.5)	1 (11.1)		5.9

^a^ Number of cases takes into account the number of participants that described memories (in detail or superficially).

**Table 3:** Simulator sickness, psychological and behavioral symptoms of dementia during reminiscence sessions.

Symptoms	Session 1 (*n* = 9)	Session 2 (*n* = 8)	Session 3 (*n* = 9)	Session 4 (*n* = 7)
	*n* (%)	*n* (%)	*n* (%)	*n* (%)
Simulator sickness ^a^				
Eyestrain	1 (11.1)		1 (11.1)	
Fullness of head	1 (11.1)	1 (12.5)		
Blurred vision		1 (12.5)		
Burping				1 (14.3)
Psychological and behavioral symptoms ^b^				
Anxiety	2 (22.2)		1 (11.1)	2 (28.6)
Agitation	3 (33.3)		1 (11.1)	2 (28.6)
Irritability	1 (11.1)	1 (12.5)	1 (11.1)	
Delusions			1 (11.1)	

^a^ Reported cases of an increase in simulator sickness (from none to slight or from slight to moderate, comparing pre and post-session). Severe simulator sickness was not reported and there was no increase from none to moderate symptoms. There was no increase in the following symptoms (which are excluded from the table): general discomfort, fatigue, headache, difficulty focusing, increased salivation, sweating, nausea, difficulty concentrating, dizzy (eyes open), dizzy (eyes closed), vertigo and stomach awareness. ^b^ Reported cases of mild/intermittent symptoms (no moderate or severe symptoms were observed). There were no cases of depression, aggression, euphoria, apathy, disinhibition and hallucinations (excluded from the table).

**Table 4:** Neuropsychiatric Inventory and EUROHIS-QOL-8 scores, pre- and post-intervention.

Measures	Pre-Intervention	Post-Intervention	*p*-Value ^a^
	Mean ± SD	Mean ± SD	
Neuropsychiatric Inventory score (0–144)	9.2 ± 5.3	9.7 ± 5.3	0.90
EUROHIS-QOL-8 score (8–40)	28.6 ± 5.9	29.2 ± 6.9	0.66

^a^ Obtained with the Wilcoxon test.
